# Crosstalk between Hydrogen Sulfide and Other Signal Molecules Regulates Plant Growth and Development

**DOI:** 10.3390/ijms21134593

**Published:** 2020-06-28

**Authors:** Lijuan Xuan, Jian Li, Xinyu Wang, Chongying Wang

**Affiliations:** Ministry of Education Key Laboratory of Cell Activities and Stress Adaptations, School of Life Sciences, Lanzhou University, Lanzhou 730000, China; xuanlj17@lzu.edu.cn (L.X.); Lijian18@lzu.edu.cn (J.L.); wangxy@lzu.edu.cn (X.W.)

**Keywords:** hydrogen sulfide, reactive oxygen species, S-sulfhydration, plant hormone, gasotransmitter

## Abstract

Hydrogen sulfide (H_2_S), once recognized only as a poisonous gas, is now considered the third endogenous gaseous transmitter, along with nitric oxide (NO) and carbon monoxide (CO). Multiple lines of emerging evidence suggest that H_2_S plays positive roles in plant growth and development when at appropriate concentrations, including seed germination, root development, photosynthesis, stomatal movement, and organ abscission under both normal and stress conditions. H_2_S influences these processes by altering gene expression and enzyme activities, as well as regulating the contents of some secondary metabolites. In its regulatory roles, H_2_S always interacts with either plant hormones, other gasotransmitters, or ionic signals, such as abscisic acid (ABA), ethylene, auxin, CO, NO, and Ca^2+^. Remarkably, H_2_S also contributes to the post-translational modification of proteins to affect protein activities, structures, and sub-cellular localization. Here, we review the functions of H_2_S at different stages of plant development, focusing on the S-sulfhydration of proteins mediated by H_2_S and the crosstalk between H_2_S and other signaling molecules.

## 1. Introduction

**Figure 1:**
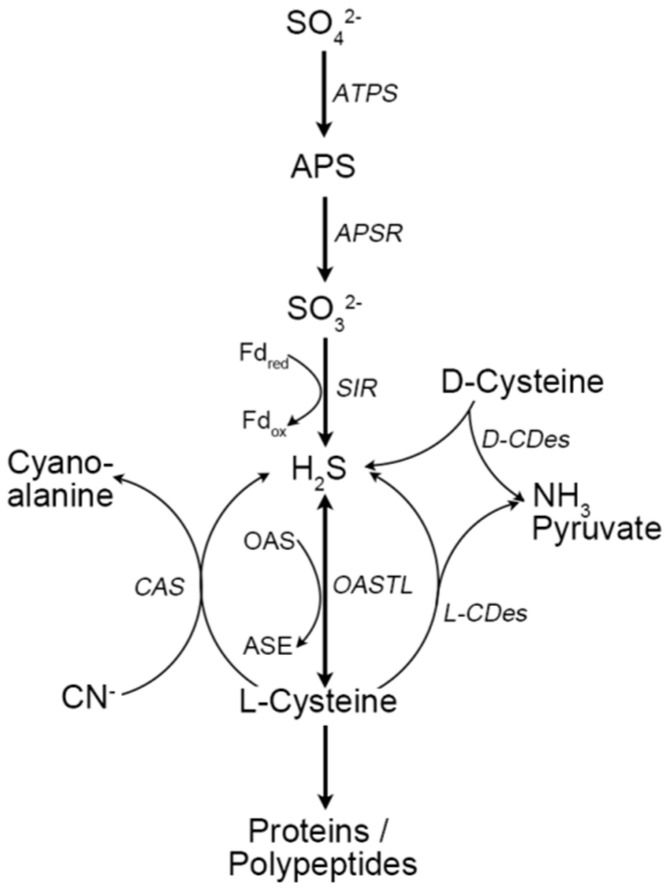
The synthesis and metabolism of H_2_S in higher plants. H_2_S is generated coincident with sulfate reduction in the plant cell. The key enzymes in H_2_S biosynthesis and metabolism include sulfite reductase (SIR), L-cysteine desulfhydrase (L-CDes), D-cysteine desulfhydrase (D-CDes), β-cyanoalanine synthase (CAS), and O-acetylserine(thiol)lyase (OAS-TL). Plants are capable of reducing activated sulfate (SO_4_^2−^) to sulfite (SO_3_^2−^), after that SIR catalyzes SO_3_^2−^ to H_2_S, with ferredoxin (Fd_red_) as the electron donor. In the presence of OAS-TL, the generated H_2_S is reversibly reduced to L-cysteine by reacting with O-acetylserine (OAS). L-CDes and D-CDes catalyze the degradation of L/D-cysteine to produce H_2_S, amine (NH_3_) and pyruvate to maintain H_2_S homeostasis. CAS, located in the mitochondria, can also catalyze the production of H_2_S, using cyanide (CN^−^) and cysteine as substrates, removing the toxin cyanogen.

Sulfur (S) is an essential element and is involved in the synthesis and metabolism of the sulfur-containing amino acids cysteine (Cys) and methionine (Met), as well as co-enzyme A, thiamine, biotin, iron-sulfur clusters, and nitrogenase. Only plants, algae, fungi, and some prokaryotes can take advantage of the inorganic sulfur (sulfate, SO_4_^2−^) naturally found in soils and incorporate it into organic forms [1]. During sulfur assimilation in plants, the SO_4_^2−^ absorbed by roots is first reduced to hydrogen sulfide (H_2_S) under the catalysis of adenosine-5′-phosphoryl sulfate reductase (APSR) and sulfite reductase (SIR) and then transformed into Cys under the catalysis of O-Acetylserine (thiol) lyase (OAS-TL). Therefore, H_2_S is an extremely important intermediate in the thio-metabolism pathway. H_2_S can also be generated from chloroplasts and mitochondria through the reduction of Cys by β-cyanoalanine synthase (CAS) and cysteine desulfhydrase (CDes) [2,3,4,5]. CAS can transform cyanide (CN^−^) and L-Cys into β–cyanoalanine and H_2_S to degrade the toxin cyanogen (Figure 1) [3,6,7]. CDes, such as L-cysteine desulfhydrase (LCD, at3g62130) [2], L-cysteine desulfhydrase 1 (DES1, at5g28030) [4], D-cysteine desulfhydrase 1 (DCD1, at1g48420), and D-cysteine desulfhydrase 2 (DCD2, at3g26115) in Arabidopsis, catalyze both L-Cys and D-Cys into H_2_S, pyruvate, and ammonia. LCD and DES1 use L-Cys as substrate and are the two pivotal enzymes in the process of endogenous H_2_S production [2].

H_2_S is a toxic gaseous molecule with the pungent odor of rotten eggs and has serious impacts on animals and plants [8]. Just 30 μmol/L H_2_S can inhibit the activity of mitochondrial cytochrome c oxidase and reduce the intensity of mitochondrial respiration by 50% [9]. Surprisingly, H_2_S also functions as a gasotransmitter, with essential roles at different stages of plant development. H_2_S interacts with other signals, such as plant hormones, other gasotransmitters, and ionic signals. H_2_S can also post-translationally modify proteins or affect secondary metabolism [10]. During seed imbibition, the endogenous H_2_S level increases in Arabidopsis under normal growth conditions [11]. When the germination of wheat seed is inhibited under copper (Cu) stress, treatment with an appropriate concentration of exogenous H_2_S (1.4 mM) promotes germination by reducing oxidative damage [12]. In addition, H_2_S induces stomatal movement of guard cells and serves as a switch in stomatal opening [13]. The application of an exogenous H_2_S donor (sodium hydrosulfide (NaHS) or GYY4137 (morpholine-4-4-methoxyphenyl)) reduces the nitric oxide (NO) accumulation induced by abscisic acid (ABA) and promotes guard cell movement to allow stomatal opening in light or darkness [14]. In Arabidopsis, mutation of *des1* leads to premature senescence of leaves [15]. In many fruits and vegetables, H_2_S treatment delays premature leaf senescence and the decay of fruits after harvest via reducing the accumulation of reactive oxygen species (ROS) [16,17] and inhibits the abscission of plant organs via increasing the content of auxin in abscission zone tissues [18]. A recent report has shown that there is a significant increase in the S-sulfhydration level of the actin proteins in an H_2_S-overproducing line, created by the over-expression of *LCD* in the Arabidopsis *O-acetylserine(thiol)lyase isoform a1* (*oasa1*) mutant (*OE LCD-5*/*oas-a1*). This increase in S-sulfhydration decreased the distribution of the actin cytoskeleton, which directly weakened actin polymerization and impaired root hair growth [19].

Here, we comprehensively review the functions of H_2_S in plant growth and development under normal or adverse environmental conditions and the mechanisms by which H_2_S influences different processes. The review focuses on both the crosstalk of H_2_S with other signals and the H_2_S-mediated S-sulfhydration of proteins.

## 2. Roles of H_2_S at Different Stages of Plant Development

### 2.1. H_2_S Promotes Seed Germination

Seed germination, the first step of the plant life cycle, is quite vulnerable to unfavorable environmental conditions [20], and several studies have addressed the concentration of H_2_S that contributes to seed germination under normal or stress conditions. For instance, when the seeds and later roots of bean, pea, wheat, and corn were exposed to 10–100 mM H_2_S solutions, their germination rate and seedling size were increased, and their germination times were shortened. After growing to maturity in soil, the total mass, roots and fruits of all H_2_S-pretreated plants were greater than the controls [21]. In imbibed seeds, the activities of L/D-CDes were stimulated and the content of H_2_S increased slightly compared with the dry seeds [11]. In the presence of hypotaurine (HT, an H_2_S scavenger) or DL propargylglycine (PAG, a DES1 inhibitor), seed germination was delayed [11], suggesting that H_2_S is indispensable in seed germination. Furthermore, metal, osmotic, and heat stresses often cause oxidative damage during seed germination. In wheat seeds inhibited by Cu, aluminum (Al), or osmotic stresses, treatment with the H_2_S donor, 1.4 mM NaHS, not only increased the content of endogenous H_2_S but also improved germination, with increased activities of amylase and esterase. Meanwhile, NaHS treatment prevented the absorption of Cu and maintained lower levels of malondialdehyde (MDA) and hydrogen peroxide (H_2_O_2_) [12,22,23]. It was concluded that H_2_S plays an important role in promoting seed germination during ionic stress by reducing oxidative damage and preventing the absorption of metal ions.

Heat stress generally suppresses seed germination by enhancing the contents of ABA, which acts through ABA-INSENSITIVE 5 (ABI5) and ELONGATED HYPCOTYL 5 (HY5), positive regulators of ABA inhibition of seed germination [24]. In maize seeds under high temperature, pre-soaking with 0.5 mM NaHS enhanced seed germination rates, sprout length, root length, and fresh weight [25]. In Arabidopsis seeds under heat stress, 0.1 mM H_2_S treatment broke the ABA inhibition on seed germination. This was shown to be due to decreased translocation of the E3 ligase CONSTITUTIVE PHOTOMORPHOGENESIS 1 (COP1) from the nucleus to the cytoplasm, causing continued degradation of ELONGATED HYPCOTYL 5 (HY5) in the nucleus. Degradation of HY5 in the nucleus inhibits ABA signaling since the transcription of *ABI5* could not be activated by HY5. H_2_S is thus potentially important in the modulation of thermotolerance of seed germination [24].

Li et al. (2012) found that soaking *Jatropha curcas* seeds with H_2_O_2_ could greatly improve the germination rate by stimulating LCD activity and H_2_S accumulation [26]. Interestingly, germination was enhanced by exogenous H_2_S but was reduced by pretreatment with an H_2_S biosynthesis inhibitor (aminooxyacetic acid, AOA). Thus, H_2_S plays a vital role in H_2_O_2_-induced seed germination in *Jatropha curcas* [26].

Together, these reports show that the content of endogenous H_2_S increases during seed germination and that exogenous NaHS treatment enhances the production of endogenous H_2_S, which in turn protects seed germination from damage by enhancing the activities of amylase and esterase, by reducing oxidative damage, by preventing the absorption of metal ions, and by repressing ABA signaling.

However, there are also reports of confounding roles for H_2_S during seed germination. In the *des1* mutant of Arabidopsis, the content of H_2_S remained unchanged after imbibition, and there was no significant difference in seed germination between wild type (WT) and *des1* under a range of temperatures (15–25 °C) and either 1 µM or 5 µM ABA [11]. In another study, when wheat TaD-CDes was ectopically overexpressed in Arabidopsis, both the transcription level and enzyme activity of D-CDes were increased, but the seed germination of TaD-CDes-expressing plants was more sensitive to ABA [27]. Therefore, the above results indicate that appropriate increase in the content of H_2_S aids seed germination under both normal and stress conditions but that endogenous H_2_S has an incompatible role with the exogenous application of H_2_S under ABA treatment, and the mechanism is not clear absolutely.

### 2.2. H_2_S Affects Formation of Lateral Roots

The development of plant root is primarily regulated by indoleacetic acid (IAA) [28]. However, recent studies have shown that H_2_S plays a significant role in the development of lateral roots by interacting with IAA, NO or H_2_O_2_ [29,30,31]. For instance, Zhang et al. (2009) reported that the application of 0.2 mM NaHS on cuttings of *Ipomoea batatas* seedlings promoted the number and the length of adventitious roots in a dose-dependent manner with increases in IAA and NO [29]. Further research showed that 1 mM NaHS pretreatment induced the up-regulation of an auxin-dependent *Cyclin Dependent Kinases (CDK)* gene (*SICDKA1*) and a cell cycle regulatory gene (*SICYCA2*) and the down-regulation of the *Kip-Related Protein 2* (*SlKRP2*), which is dependent on NO signaling [32]. The gene expression induced by H_2_S could be blocked by an IAA transport inhibitor (N-1-naphthylphthalamic acid; NPA) or a NO scavenger [2-(4-carboxyphenyl)-4,4,5,5 -tetramethylimidazoline-1- oxyl-3- oxide; cPTIO], indicating that the lateral root development promoted by H_2_S is also dependent on NO and IAA signaling through regulation of *SICDKA1*, *SICYCA2* and *SlKRP2* [29]. Moreover, 1 mM NaHS treatment upregulated the *respiration burst oxidase homologous (RBOH1)* transcript, resulting in the overproduction of H_2_O_2_, contributing to lateral root formation in tomato. However, when plants were co-treated with an H_2_O_2_ scavenger (dimethylthiourea; DMTU) and an inhibitor of NADPH oxidase (diphenylene iodonium; DPI), the lateral root formation induced by NaHS was impaired, and the up-regulation of *SlCYCA2;1*, *SlCYCA3;1*, and *SlCDKA1* and the down-regulation of *SlKRP2* induced by H_2_S was suppressed [31]. Therefore, it can be concluded that H_2_S treatment upregulated the *RBOH1* transcript and promoted the production of H_2_O_2_, which stimulated NO and IAA signaling through regulation of the expression of *SICDKA1, SICYCA2, SlCDKA1* and *SlKRP2*, leading to lateral root formation.

Methane (CH_4_) is another gaseous compound that may transmit signals. Recent studies have shown that CH_4_ plays an important role in some plant physiological processes, such as responses to water, salt and heavy metal stressors [33,34,35]. In addition, CH_4_ was also found to participate in root organogenesis, an activity that might be related to NO [36]. Kou et al. (2018) discovered that CH_4_ treatment increased the expression levels of *L-CDes* genes and the endogenous H_2_S content, which then promoted adventitious root development in cucumber with the up-regulation of genes related to cell division, namely *CsDNAJ-1*, *CsCDPK*, *CsCDPK5*, and *CsCDC6*, to auxin signaling, namely *CsAux22D-like* and *CsAux22B-like*, and that this response was disrupted by the presence of the H_2_S scavenger HT or the DES1 inhibitor PAG [37]. Similar results were reported in tomatoes [38]. Further, the lateral roots of the *Atdes1* mutant showed defects in the presence of CH_4_ in Arabidopsis [38]. All these data demonstrated that *DES*-dependent H_2_S signaling plays a major role in CH_4_-triggered lateral root formation.

Chen et al. (2014) found that selenium (Se) stress inhibited root growth in *Brassica napus* by suppressing the expression of most of the LCD and DCD homologues [30]. Pretreatment with 0.5 mM NaHS alleviated the inhibitory effect of Se on root growth by partly restoring the endogenous H_2_S content in roots and reducing the accumulation of ROS by increasing the content of glutathione (GSH), suggesting that both H_2_S and GSH are involved in the regulation of lateral root growth under stress through antioxidation [30].

However, in some other studies, high levels of H_2_S (100–500 μM) changed root development by inhibiting auxin transport and thus altering the polar subcellular distribution of the PIN proteins, which is an actin-dependent process [39]. NaHS treatment (100 μM) and the overproduction of endogenous H_2_S in the *OE LCD-5/oasa1* line significantly increased the S-sulfhydration level of actin-2 and decreased the distribution of actin cytoskeleton in root cells, which directly weakened the aggregation of actin and reduced the root hair density of Arabidopsis [19]. Overexpression of D-CDes further inhibited root growth under ABA treatment [27]. Based on the above studies, it is speculated that the concentration of H_2_S may vary in different plants. The appropriate concentration of H_2_S promotes the formation of adventitious roots by affecting the expression of cell division-related genes and auxin signaling-related genes or by reducing the accumulation of ROS induced by stress. In Arabidopsis, increasing the endogenous H_2_S levels, either through treatment with high concentration of NaHS (100 μM) or through overexpression of a *CDes* gene, to a harmful level that affects lateral root development through the S-sulfhydration of actin-2, a posttranslational modification.

### 2.3. H_2_S Regulates Plant Stomata Movement and Photosynthesis

Stomata are the channels that allow the exchange of gas and water between plants and the environment, and their opening and closing regulate the important physiological processes of photosynthesis and transpiration, thus affecting the growth and development of plants [40]. Stomatal movement and photosynthesis are most often influenced by environmental factors—including light, temperature, and water-and regulated by plant hormones, such as ABA, jasmonic acid (JA), or ethylene (ET). Other important signaling components influencing stomatal movement are Ca^2+^, NO, and H_2_O_2_ [41,42,43,44,45].

At present, a number of studies have confirmed that H_2_S, as a gaseous signaling molecule, also regulates stomatal movement of guard cells [13,46]. For instance, under normal conditions, 0.01 mM H_2_S treatment improved photosynthesis by increasing stomatal aperture and density and reducing photorespiration in rice and *Spinacia oleracea* [16,47]. In tall fescue, 500 μM H_2_S increased photochemical efficiency and antioxidant enzyme activities while reducing the levels of H_2_O_2_ and MDA under low-light stress conditions [48]. In blueberry seedlings, exogenous 500 μM H_2_S alleviated low temperature stress by maintaining the content of chlorophyll, carotenoids, and the osmotic regulator proline and by reducing photosynthetic inhibition and membrane peroxidation [49].

In guard cells, other studies have shown that both ET and ABA could increase L/D-CDes activity, resulting in an increase of H_2_S content [50,51]. In *Vicia faba* L. and Arabidopsis, the application of an H_2_S synthesis inhibitor (AOA), NO scavenger (cPTIO), or NO synthesis inhibitor (Na_2_WO_4_) suggested that H_2_S was located downstream of the NO signal that regulates ET-induced stomatal closure [51,52,53]. In addition, D-CDes overexpression accelerated ABA-induced stomatal closure by up-regulating the expression of ABA-responsive genes [27], while the mutation of *des1* blocked ABA-induced stomatal closure through the signaling pathway of LONG HYPOCOTYL1 (HY1, a member of the heme oxygenase family) [54]. Further investigation revealed that, under the induction of ABA, the Cys44 and Cys205 residues of DES1 were persulfidated by H_2_S_,_ and DES1 activity was also rapidly activated, resulting in a large amount of intracellular H_2_S accumulation in a short period of time. Furthermore, this sustainable H_2_S accumulation contributed to the S-sulfhydration of the NADPH oxidase RBOHD at Cys825 and Cys890, which could then stimulate a large amount of ROS production. Simultaneously, excessive intracellular production of ROS could induce stomatal closure and negatively regulate the degree of S-sulfhydration of DES1 and RBOHD, and thus played a role in feedback inhibition of ABA signaling [55]. Additionally, the accumulation of H_2_S induced by ABA could also mediate the S-sulfhydration of SNF1-RELATED PROTEIN KINASE 2.6 (SnRK2.6), which in turn positively regulates ABA signaling to induce stomatal closure [56]. Therefore, during ABA-induced stomatal closure, H_2_S, on the one hand, activates ABA signaling via the S-sulfhydration of SnRK2.6 and, on the other hand, is a feed-back regulator of ABA signaling via the S-sulfhydration of RBOHD, which then induces stomatal movement.

Inconsistence with the above results, the application of an exogenous H_2_S donor, 200 μM NaHS or 200 μM GYY4137, caused guard cell to open stomata in light or darkness by reducing the NO accumulation induced by ABA in *Capsium anuum* and Arabidopsis [14,57]. As discussed above, it is speculated that a low concentration of H_2_S participates in regulating stomatal closure induced by drought, ABA, or ET and enhances photosynthesis by acting with NO, H_2_O_2_ or persulfidation-based modification of proteins. However, a high concentration of H_2_S can prevent stomatal closure. This paradox is emblematic of the double-sided effect of H_2_S.

### 2.4. H_2_S Delays Plant Senescence

Plant senescence is an actively programmed cell death (PCD), which not only occurs naturally in the plant life cycle during times such as leaf senescence, fruit ripening and abscission, but also when a plant is subjected to darkness, drought, disease, low temperature and other stresses [58]. At the molecular level, plant senescence is mainly regulated by plant hormones—including cytokinin (CTK), gibberellin (GA), ET, brassinolide (BR), salicylic acid (SA), and JA—by *senescence-associated genes* (*SAGs*) and by WRKY family transcription factors [59]. However, recent research revealed that H_2_S also participates in the regulation of plant senescence.

#### 2.4.1. H_2_S Delays Leaf Senescence

Leaf senescence is an important developmental process, which involves a variety of metabolic changes related to macromolecular degradation, recycling nutrients back to the main plant body [60]. In *S. oleracea* seedlings, the senescent leaves had higher H_2_S levels than the new leaves, indicating that H_2_S may also be involved in the regulation of plant senescence [16]. Zhang et al. (2011) showed that the flower and shoot explants from *Gossypium* and *Salix*, treated with 0.6 mM and 0.2 mM NaHS, respectively, increased the activities of catalase (CAT), superoxide dismutase (SOD), and APX and kept the low levels of MDA, H_2_O_2_ and superoxide anion (•O_2_^−^), which resulted in prolonging fresh cut flowers and one-year-old shoots [61]. In detached leaves of Arabidopsis, 0.5 mM H_2_S inhibited chlorophyll degradation by regulating the dark-dependent response, and actively regulated the expression of *SAGs*, such as *SAG1* and *SAG21*, in a manner dependent on *S-nitrosoglutathione reductase 1* (*GSNOR1*) under long dark condition [62]. The leaves in the Arabidopsis *des1* mutant showed premature senescence and higher expression of *SAG1*, *SAG21* and related transcription factors compared to WT. Remarkably, senescence-associated vesicles, related to cell autophagy, were detected in mesophyll protoplasts in the *des1* mutant, and *DES1* deficiency stimulated the accumulation and lipidation of autophagy related protein-8 (ATG8) [15]. Moreover, treatment with an H_2_S donor, NaHS, or sodium sulfide (Na_2_S), negatively regulated autophagy in Arabidopsis in a way that was unrelated to ROS or nutrient deficiency [63,64]. Thus, H_2_S might regulate plant senescence by reducing ROS accumulation and chlorophyll degradation, positively regulating *SAG* genes expression, and negatively regulating autophagy. Nevertheless, the mechanism by which H_2_S regulates autophagy is unclear. There is some evidence that H_2_S regulation of autophagy might be related to the persulfidation of autophagy-related proteins (ATGs), such as ATG18a, ATG3, ATG5, ATG4, or ATG7 [15,63,65,66].

#### 2.4.2. H_2_S Delays the Postharvest Maturation of Fruits

Postharvest maturation of fruits and vegetables is also a type of senescence. H_2_S treatment positively regulates certain physiological aspects of ripening, such as color metabolism, softening, and postharvest decay during storage, suggesting that H_2_S might regulate aging to protect the ripening and quality changes in various fruits and vegetables [17]. In addition, H_2_S can eliminate ROS in harvested produce by promoting the activities of antioxidant enzymes, through synergism (NO) or antagonism (ET) with other molecules, and by regulating the expression of *SAGs* related to protein and chlorophyll degradation in order to maintain the integrity of membranes and to slow senescence [67]. In softening kiwifruit, 45–90 μM NaHS treatment up-regulated the activities of protective enzymes, such as SOD and CAT, and down-regulated the levels of ROS and ET during storage [68]. Moreover, H_2_S contributed to the maintenance of firmness and the soluble solids content, affecting the expression of related genes, and to the protection of the integrity of the cell wall and modulation of ET signal transduction [69]. During postharvest storage of tomato fruit, H_2_S acts as an antagonist to ET, coordinates antioxidative enzymes, and reduces the production of •O_2_^−^, MDA, and H_2_O_2_ [70]. H_2_S has a significant role in postharvest fruit biology, through establishing crosstalk with ET, ROS, NO, oxidative stress signaling, sulfate metabolism, and post-translational modification of proteins [71]. Therefore, all of the above studies indicate that H_2_S delays postharvest maturation of fruits mainly by enhancing their antioxidant capacity to reduce the production of •O_2_^−^, MDA, and H_2_O_2_ and by establishing crosstalk with NO and ET signaling pathways. It is speculated that H_2_S can be used to delay crop aging for increasing crop yield and for keeping fruits and vegetables fresh during storage and transport.

#### 2.4.3. H_2_S Inhibits Organ Abscission in Plants

Abscission in plants refers to the process by which some organs, including leaves, flowers, fruits, seeds, and petioles, grow to a certain extent and then are removed naturally from the plant itself. Normal organ abscission is often associated with maturation and senescence [72]. For instance, most fruits undergo abscission during ripening, and petals wither and fall from flowers after pollination and fertilization [73]. Abnormal organ abscission also occurs when plants encounter unfavorable environmental conditions or are damaged by diseases or insects [74,75]. Numerous experiments have shown that plant hormones, such as auxin, ET and SA, are involved in regulating organ abscission in plants [76]. ET is a pivotal abscission inducer and has an indispensable role at different stages of abscission, such as the initiation and progression of floral and organ abscission [77,78]. Furthermore, ET is associated with INFLORESCENCE DEFICIENT IN ABSCISSION (IDA)-mediated floral organ abscission through regulation of the transcription of DNA binding with one finger 4.7 (AtDOF4.7), which can directly impair the expression of the abscission-related gene *ARABIDOPSIS DEHISCENCE ZONE POLYGALACTURONASE 2 (ADPG2)* in Arabidopsis [79]. Liu et al. (2020) demonstrated that H_2_S also participates in ET-induced petiole abscission of tomato [18]. The research showed that H_2_S treatment could delay abscission of the tomato petiole, but the situation was reversed when the plants were exposed to an H_2_S scavenger. Moreover, H_2_S treatment reduced the enzymatic activities that modify the cell wall. Along with the expression levels of IAA/AUX family genes (*SlIAA3* and *SlIAA4*), the transcription of genes in the IAA-amino acid conjugate hydrolase (ILR) family (*ILR-L3* and *ILR-L4*) were found to be up-regulated and down-regulated, respectively, in the abscission process, suggesting that H_2_S prevented ET-induced petiole abscission by increasing the content of auxin in abscission zone tissues [18]. Additionally, Hideo et al. (2019) reported that D-Cys, as a physiologically relevant substrate, participates in the process of root abscission and that exogenous application of H_2_S chemical donors or polysulfides can positively induce abscission to cope with environmental stimuli in the water fern *Azolla* [80]. Therefore, H_2_S also plays a positive role in ET-induced organ abscission by regulating the transcription of IAA-related genes and by promoting the accumulation of auxin in abscission zone tissues.

## 3. Mechanism by which H_2_S Regulates Plant Growth and Development

### 3.1. Crosstalk of H_2_S with Plant Hormones

Phytohormonea are indispensable regulators of plant growth and development. A large number of studies have showed that H_2_S closely interacts with the plant hormones ABA, ET, auxin, SA, GA, and JA during plant growth and development under normal or stress conditions.

#### 3.1.1. Crosstalk of H_2_S with Abscisic Acid

ABA plays important roles in many physiological processes of plants, such as maintaining seed dormancy, promoting plant senescence, and even responding to drought stress [81]. A recent study showed that ABA could activate the gene expression and enzyme activities of *LCD/DES1*, which are responsible for the synthesis of H_2_S [13]. On the other hand, exogenous H_2_S treatment accelerated stomatal closure induced by ABA in *Vicia faba*, *Arabidopsis thaliana*, and *Impatiens walleriana*, suggesting that H_2_S may participate in the ABA-induced stomatal closure [46]. Further analysis showed that ectopic expression of *D-CDes* from wheat (*TaD-CDes*) in Arabidopsis makes plants more sensitive to ABA, which means that ectopic expression of *TaD-CDe* amplifies the stomatal closure and root shortening and further delays the seed germination and cotyledon greening induced by ABA. Simultaneously, TaD-CDe plants showed up-regulation of the ABA receptor PYR1; the ABA responsive element-binding factors ABF2 and ABF4; and the ABA negative regulators ABI1, ABI2, HAB1, and HAB2, and down-regulation of ABA-induced SNF1-related protein kinases (SnRK2.2, SnRK2.3, and SnRK2.6) [27]. Moreover, the accumulation of H_2_S induced by ABA in turn activates the activity of SnRK2.6 by the S-sulfhydration of SnRK2.6 at Cys131 and Cys137, which enhances the interaction of SnRK2.6 with ABF2. Thereby, H_2_S plays a positive role in the regulation of ABA-induced stomatal closure through mediating the S-sulfhydration of SnRK2.6 [56]. Another study demonstrated that H_2_S mediated the S-sulfhydration of DES1 at Cys44 and Cys205, which is stimulated by ABA and positively activates DES1 activity, leading to further accumulation of H_2_S [55]. However, excessive production of ROS in turn inhibits the S-sulfhydration of DES1 and RBOHD, forming a feedback regulation mechanism to control ABA signaling [55]. On the other hand, pretreatment with an ATP-binding cassette (ABC)-transporter inhibitor (glibenclamide), an H_2_S scavenger (HT) or an H_2_S synthesis inhibitor (PAG), blocks ABA signaling, suggesting that the regulation of ABC transporters play a critical role in the signaling transduction of ABA-dependent stomatal closure mediated by H_2_S [46]. Taken together, we can conclude that H_2_S activates ABA signaling through mediating the S-sulfhydration of SnRK2.6 and that higher levels of H_2_S tamps down ABA signaling by mediating the S-sulfhydration of RBOHD, leading to an increase in ROS, thereby balancing the ABA signal. ATP also plays an important role in the cross-talk between H_2_S and ABA [46].

In order to study the close relationship between H_2_S and ABA under drought stress, the mutants *lcd*, *aba3*, and *abi1* were studied. Compared with WT, the *lcd* mutant showed a weakened response to ABA-induced stomatal closure and was more sensitive to drought stress with the decrease of expression of ion-channel coding genes for Ca^2+^ and outward-rectifying K^+^ channels, and, conversely, an increase of inward-rectifying K^+^ and anion channels. In both the *aba3* and *abi1* mutants, the stomatal aperture was increased with the decrease of *LCD* expression and H_2_S production rate. Remarkably, NaHS treatment rescues all the above defects, implying that H_2_S is an important mediator in the ABA- regulated stomatal response to drought through ion channels [82]. In addition, Li et al. (2016) found that ABA treatment increased the activity of LCD in tobacco cells under high temperature and that application of NaHS enhanced the heat tolerance induced by ABA by alleviating the increase in MDA content and electrolyte leakage [83]. This effect of exogenous H_2_S or ABA treatment was weakened by the addition of an H_2_S scavenger or a specific inhibitor of H_2_S biosynthesis, suggesting that there is a synergistic effect between H_2_S- and ABA-mediated heat resistances of tobacco suspension-cultured cells [83]. More research discovered that application of H_2_S promoted the accumulation of the E3 ligase COP1 in the nucleus, resulting in the degradation of HY5 and a decrease in *ABI5* expression, which lead to a decrease of ABA content and enhanced seed germination under high temperatures [24]. Therefore, it is speculated that H_2_S may cooperate with ABA signaling to enhance the tolerance of plants to drought stress by activating Ca^2+^ signaling and inward-rectifying K^+^ channels. Under heat stress, H_2_S cooperates with ABA signaling to promote seed germination and growth by reducing oxidative damage and regulating the expression of ABA-related genes.

#### 3.1.2. Crosstalk of H_2_S with Ethylene

ET has many roles, including inducing stomatal closure. In Arabidopsis, ET significantly affects the transcription of *AtD-CDes*. Similarly,1-aminocyclopropane-1-carboxylic acid (ACC), a precursor of ET, treatment increases the content of H_2_S and the activities of D/L-CDes [51]. Although inhibitors of D/L-CDes alone cannot inhibit stomatal closure, they do significantly inhibit ACC-induced stomatal closure. Furthermore, *L/D-Cdes* overexpression plants are more sensitive to ET. Thus, H_2_S may be located downstream of ET and work synergistically with ET to induce stomatal closure, similar to its interaction with ABA [51,52]. However, further research revealed that when the NO content decreased, the ET induction of H_2_S and of L/D-CDes activities was reduced. The inhibition of H_2_S synthesis had no effect on the accumulation of NO and the activity of nitrate reductase (NR). Furthermore, ET induced NO synthesis but failed to enhance stomatal closure in the NO-related mutants *atnia1* and *nia2*, indicating that H_2_S enhances ET-induced stomatal closure under the guidance of NO [52]. Equally, in *Vicia faba*, H_2_S is a key participant in ET-induced stomatal closure downstream of NO [53], but their interaction mechanism is not clear.

ET-fumigation promotes the ripening of fruits with increases in the content of ROS and MDA. Li et al. (2017) found that H_2_S treatment could effectively alleviate ET-induced fruit softening when fumigated kiwifruit with both ET and H_2_S while increasing the levels of ascorbic acid, titratable acid, starch, and soluble protein and reducing sugar [84]. In addition, ET and H_2_S treatment enhanced the activities of antioxidant enzymes (CAT, APX) and reduced the oxidative stress of the fruits. Further research showed that H_2_S inhibited the expression of ET synthesis-related genes and decreased the expression of Cys protease genes [84]. In addition to fruit ripening, ET positively regulates organ abscission. Liu et al. (2020) recently showed through H_2_S-ET co-treatment that H_2_S inhibited the up-regulation of ET synthesis and signal transduction genes, including *ACS6*, *ACO1*, *ACO4*, *ERF1*, and *ETR4*, eventually resulting in the suppression of ET-induced petiole abscission in tomato [18]. Together, these experiments show that, during fruits ripening, senescence and organ abscission, H_2_S antagonizes the effects of ET by reducing oxidative stress and reducing the expression of ET-related genes and ET synthesis, thereby suppressing the ET signaling.

What is the relationship between H_2_S and ET under stress condition? Jia et al. (2018) revealed that an H_2_S scavenger (HT) or synthesis inhibitor (PAG) could eliminate the effect of ET or osmotic stress on stomatal closure, indicating that H_2_S is a necessary downstream factor of ET-induced stomatal closure under osmotic stress [50]. However, under hypoxia, NaHS pretreatment inhibited the activity of ACC oxidase (ACO), a key enzyme in ET biosynthesis [85]. Moreover, it was documented that H_2_S reduced ethylene synthesis by inhibiting the transcription of *LeACO* genes and restraining the activities of LeACO1 and LeACO2 by inducing the S-sulfhydration of LeACO1 at Cys60 in a dose-dependent manner [50]. In short, these data show that the ET-induced H_2_S signal has a negative regulatory effect on ET biosynthesis through mediating S-sulfhydration of ACO.

#### 3.1.3. Crosstalk of H_2_S with Auxin

Auxin affects many stages of plant growth and development, coordinating the adaptation of plant growth and morphology to environmental conditions [86]. During lateral root development, NaHS treatment rapidly increases the content of auxin and promotes the number and length of adventitious roots, showing that there may be also a close cross-talk between H_2_S and auxin [29]. Auxin normally inhibits organ abscission, and further investigation showed that the IAA/auxin family genes (*IAA3* and *IAA4*) are often up-regulated by H_2_S [18]. In cuttings from sweet potato seedlings and excised willow shoots and soybean seedlings, both the IAA polar transport inhibitor NPA and the NO scavenger (cPTIO) can disturb the formation of root system mediated by H_2_S. It is speculated that H_2_S acts as upstream of NO and IAA to promote root hair development or to restrain organ abscission [29]. However, auxin-insufficiency weakened DES1 activity and reduced the content of H_2_S in tomato. Both NAA and NaHS can counteract the effects of auxin deficiency on *SlDES1* transcription, DES1 activity and endogenous H_2_S content and can rescue the stimulation of lateral roots induced by auxin depletion [32]. Simultaneously, NaHS- or NAA-induced up-regulation of the cell cycle regulatory genes *SlCDKA;1* and *SlCYCA2;1* and down-regulation of *SlKRP2* were reversed after exposure to the scavenger HT, suggesting that H_2_S might be downstream of auxin to promote the formation of lateral roots [32]. These data suggest that there may also be feedback regulation between H_2_S and auxin during plant growth and development, in which H_2_S can up-regulate the transcription of IAA family genes, and IAA can also affect the *DES1* expression and DES1 activity.

During the plant response to pathogen, the expression of *auxin signaling F-box protein 1* (*AFB1*), *AFB2*, and *AFB3* are negatively regulated by H_2_S [87]. Furthermore, cold stress promoted the accumulation of H_2_S and also triggered the endogenous IAA system. Application of NaHS significantly increased the activity of favin monooxygenase (FMO) and the relative expression of the FMO-like protein *YUCCA2* in cucumber seedlings, which in turn increased the level of endogenous IAA and improved cold tolerance, seen as decreases in electrolyte leakage and accumulation of ROS and increases in expression of genes and enzyme activities related to photosynthesis. Application of IAA or removal of H_2_S had little effect on the signaling of the other molecule, but the IAA polar transport inhibitor NPA inhibited H_2_S-induced cold tolerance and defense gene expression [88]. IAA participates in H_2_S-induced stress tolerance in plants as a downstream signaling molecule, while H_2_S promotes auxin signal transduction by regulating the expression of auxin-related genes and the synthesis of auxin, thereby enhancing the plant tolerance to adverse environmental conditions.

#### 3.1.4. Crosstalk of H_2_S with Gibberellin

GA can regulate many aspects of plant growth and development, such as seed germination, leaf expansion, and flowering [89]. During seed germination, GA can stimulate the synthesis of α- amylase and some secreted hydrolases to break seed dormancy. H_2_S significantly enhances the activity of β-amylase and accelerates the germination of barley seeds with or without GA, although the survival rate of cells without GA is higher than those with GA. It is speculated that at the early stage of seed germination, the activation of β-amylase by H_2_S is ahead of the activation of α-amylase by GA, both of which can then degrade starch and provide sugar for seedling growth and development [90]. In the wheat aleurone layer, GA accelerates PCD, and during these, both the activity of LCD and the production of H_2_S are reduced [91]. Interestingly, application of NaHS not only inhibits the production of endogenous H_2_S, but also alleviates the PCD induced by GA. It was speculated that this reversal is related to GSH because NaHS causes an increase of endogenous GSH content, and the alleviation of NaHS-mediated PCD is eliminated by an inhibitor of GSH synthesis [91]. Therefore, the interaction between H_2_S and GA is likely indirect through the regulation of GSH homeostasis.

#### 3.1.5. Crosstalk of H_2_S with Salicylic Acid

The phenolic compound SA widely exists in plants, can be transported in the phloem, and plays multiple roles, such as improving disease resistance, drought resistance and heat resistance [92]. Li et al. (2015) discovered that SA pretreatment enhances the activity of LCD and contributes to the accumulation of endogenous H_2_S during heat tolerance response of maize seedlings [93]. The heat resistance induced by SA is enhanced by the addition of NaHS and decreased by the addition of an H_2_S-synthesis inhibitor (PAG) or scavenger (HT). However, there was no significant effect on key enzymes of SA biosynthesis and endogenous SA content. In addition, pretreatment with SA-biosynthesis inhibitors (paclobutrazol, PAC and 2-aminoindan-2-phosphonic acid, AIP) do not affect the heat tolerance induced by NaHS [93]. These results indicate that H_2_S is located downstream of SA and works with SA to induce plant resistance to heat stress.

#### 3.1.6. Crosstalk of H_2_S with Jasmonate

JA is an important endogenous regulator in higher plants, especially as an environmental signaling molecule, and both regulates plant growth and development and mediates plant defense response to biotic and abiotic stresses [94,95]. JA and JASMONATE INSENSITIVE (JIN/MYC) transcription factors are key factors in regulating stomatal development in Arabidopsis [96]. A recent experiment suggested that the removal of H_2_S increased the number of stomata inhibited by JA, while the application of NaHS alleviated the stomatal inhibition in the JA-signaling-deficient *myc234* mutant. H_2_S reduces the expression of stomate-associated genes and blocks key components of the stomatal signaling pathway, such as TOO MANY MOUTHS (TMM), STOMATAL DENSITY AND DISTRIBUTION1 (SDD1), and SPEECHLESS (SPCH). Interestingly, mutation of *LCD* increased stomatal density and index values, and an H_2_S synthesis inhibitor (HT) counteracts the JA-mediated reduction of stomatal density [97]. All of these data confirm that H_2_S is located downstream of JA and cooperates with JA to negatively regulate stomatal development.

### 3.2. Crosstalk between H_2_S and Other Gasotransmitters

H_2_S is the third known gaseous signaling molecule, along with carbon monoxide (CO) and NO. There are many similarities between these three molecules in their physiological functions in plants, such as regulating growth, enhancing the response of plants to various adversities, and improving the antioxidation capacity, and many close interactions between their signaling pathways.

#### 3.2.1. Crosstalk between H_2_S and NO

During root organogenesis, IAA, H_2_S, and NO all promote root hair growth in *Ipomoea batatas* in a dose-dependent manner, as shown by the application of the H_2_S donor NaHS, the NO donor sodium nitroprusside (SNP) and IAA [29]. Furthermore, both the NO scavenger cPTIO and the IAA transport inhibitor NPA could inhibit H_2_S-induced root hair growth. Interestingly, an H_2_S scavenger also inhibits the lateral root formation induced by NO, but not by IAA, indicating that only H_2_S and NO might be interdependent, although both NO and IAA are involved in the adventitious root formation induced by H_2_S [29]. Moreover, Zhang et al. (2017) found that a high level of NaHS treatment inhibited the growth of the primary root, which was accompanied by the accumulation of ROS and NO and activation of MITOGEN-ACTIVATED PROTEIN KINASE 6 (MPK6) [98]. Further studies showed that ROS was required for the generation of NO inducted by H_2_S, and that this induction was mediated by MPK6. Moreover, the *respiration burst oxidase homologous* (*rbohd/f*) mutant and NO biosynthesis-related mutants (*nial-2/2-5* double mutant and *noa1*) were less sensitive to NaHS, and the inhibition of NaHS on the growth of root was reduced by the NO scavenger cPTIO. These results indicate that ROS-MPK6-NO signaling mediates the inhibitory effect of high levels of H_2_S on root growth [98].

From the previous discussion of crosstalk between H_2_S and ET, we know that H_2_S may be a signaling molecule downstream of NO in ET-induced stomatal closure [52,53]. However, Lisjak et al. found that H_2_S causes stomatal opening in Arabidopsis and *Capsicum anuum*, when plants are treated with an H_2_S donor (NaHS) and a slow-release H_2_S donor molecule (GYY4137) [14,57]. Moreover, both donor molecules reduced NO accumulation caused by ABA treatment of leaf tissue [14,57]. These results suggest that the adjustment of both H_2_S and NO affects the sensitivity of stomatal movement. In the *gsnor1* mutant (which normally clears SNO to prevent NO signal transmission), the positive effect of H_2_S on *SAGs* was weakened in the dark [62], indicating that H_2_S signaling during the regulation of plant senescence depends on NO signaling [99]. Proteomic studies have also found that sites in proteins that can be S-nitrosylated by NO can also be S-sulfhydrated by H_2_S [100]. Therefore, NO and H_2_S, may compete with each other through the post-translational modification of proteins to regulate plant growth and development.

Under adverse conditions, both H_2_S and NO are important signaling molecules, but their crosstalk relationship needs to be sorted out. Recent research revealed that both application of the H_2_S donor NaHS and the NO donor SNP improved the survival rate of plants under heat stress because of reduced electron leakage accumulation of MDA, and improved antioxidant capacity [101,102]. In maize under heat stress, SNP pretreatment increases the activity of LCD, inducing the accumulation of endogenous H_2_S [101]. The application of NaHS and GYY4137 enhances the heat resistance induced by SNP, but this is eliminated by an H_2_S scavenger. Therefore, H_2_S might be a downstream signaling molecule during NO-induced heat tolerance in maize seedlings [101]. However, in strawberry during the early stage of exposure to high temperature, the application of NaHS reduced NO content, enhancing the tolerance to the heat stress [103].

During Al stress, NO is also a negative regulator [104]. H_2_S alleviates the inhibition of Al on Arabidopsis elongation by enhancing the activity of antioxidant enzymes and reducing ROS damage. In rice, H_2_S increases Al transport into vacuoles and reduces the content of NO in roots [105,106]. Therefore, it is hypothesized that H_2_S interacts with NO signaling to improve Al and heat tolerance of plants by reducing the content of NO and oxidative damage.

Hypoxic conditions, when O_2_ is lacking, often cause a ROS burst. Group VII ET-responsive factors (ERFVII) sense hypoxia and then initiate the hypoxia response. NO is required for the destabilization of ERFVII [107]. H_2_S can also enhance tolerance to hypoxia by removing the accumulated ROS and increasing the transcription of hypoxia-responsive genes (*ADH*, *CRT1*, *GS*, and *CYP51*) [85,108]. In maize seedling root tips, pretreatment with SNP enhanced the activity of key H_2_S metabolic enzymes (LCD, CAS, OAS-TL) and the accumulation of endogenous H_2_S under hypoxia, but these effects were reversed by cPTIO. Application of an H_2_S synthesis inhibitor (HA) and an H_2_S scavenger (HT) canceled out the increased survival rate induced by SNP [108]. Therefore, under adverse conditions, NO and H_2_S work interdependently to remove accumulated ROS and enhance the stress tolerance of plants.

#### 3.2.2. Crosstalk between H_2_S and CO

Although CO is also an important signaling molecule, there are relatively few studies on any crosstalk between CO and H_2_S. During root development, Heme Oxygenase-1 (HO-1), which catalyzes the production of CO, acts downstream of the auxin signaling pathway, leading to the formation of adventitious roots of cucumber [109]. Further analysis found that the addition of CO and H_2_S could also promote adventitious root formation in cucumber [110]. In pepper, NaHS induced both the *CsHO-1* gene and CsHO-1 protein expression in a time-dependent manner. The application of ZnPPIX, a specific inhibitor of HO-1, could reverse the formation of adventitious roots induced by NaHS. However, the addition of an H_2_S scavenger (HT) could not alter the effect of CO on adventitious root formation [110]. This indicates that H_2_S may play a specific role upstream of CO in the formation of adventitious roots and may promote the production of CO, which then stimulates the formation of lateral roots.

### 3.3. Crosstalk of H_2_S with Ionic Signals

#### 3.3.1. Crosstalk of H_2_S with Ca^2+^

The Ca^2+^ is one of the most important nutrient elements in plants. Ca^2+^ functions to maintain the stability of the cell wall, cell membrane and membrane binding proteins, but is also an important signaling molecule and participates in the regulation of cell homeostasis, plant growth and stress responses.

The application of exogenous NaHS increases the intracellular Ca^2+^ content under both hypoxia and heat stress [108,111]. In the suspension culture cells of tobacco, exogenous Ca^2+^ and its ionophore A23187 significantly enhances the high temperature tolerance induced by NaHS. On the other hand, the heat tolerance induced by H_2_S could be weakened by a Ca^2+^ chelating agent, the plasma membrane channel blocker La^3+^, or the calmodulin antagonist chlorpromazine or trifluoperazine. This suggests that the H_2_S-induced thermostability requires the participation of Ca^2+^, which acts as a downstream molecule, at least in tobacco suspension cells [111]. However, the application of Ca^2+^ or calmodulin (CaM), a calcium ion receptor, activates the activity of DES1 and induces the accumulation of endogenous H_2_S in tobacco suspension culture cells, and the application of a Ca^2+^ chelator or CaM antagonists reduces DES1 enzyme activity and H_2_S content. All of these increases induced by Ca^2+^/CaM, in DES1 activity, H_2_S content, and heat tolerance are enhanced by the H_2_S donor NaHS or weakened by H_2_S synthesis inhibitors or an H_2_S scavenger. Therefore, during the heat stress response process, the H_2_S and Ca^2+^ signals may be interdependent [112].

Similarly, chromium (Cr^6+^) stress activates endogenous H_2_S synthesis and Ca^2+^ signaling transduction. The damage caused by Cr^6+^ stress is greatly alleviated by application of H_2_S and Ca^2+^ alone or in combination, with the combined addition more effective. In contrast, the induced stress was intensified by treatment with an H_2_S synthesis inhibitor or Ca^2+^ chelators. This illustrated the synergistic effect of H_2_S and Ca^2+^ under Cr^6+^ stress [113]. Furthermore, during Cr^6+^ stress, the metallothionein (encoded by *MT3A*) and phytochelatin (synthesized by phytochelatin synthase, PCS) bind the heavy metal to provide protection to the plant cells. The upregulation of *MT3A* and *PCS*, regulated by Ca^2+^, is dependent on H_2_S signaling [113].

Calcium dependent protein kinases (CDPK) are important protein kinases in plant signal transduction. CDPK can be activated directly by combination with Ca^2+^. An activated CDPK protein can be phosphorylated to amplify Ca^2+^ signaling. Experiments in Arabidopsis revealed that both H_2_S and CDPK are involved in the cadmium (Cd) stress response through the alleviation of the oxidative stress. Moreover, mutation of *CDPK* or treatment with the CDPK inhibitor TFP reduces LCD enzyme activity and H_2_S content. In the *cdpk3* mutant, H_2_S increases the transcription of Cd stress-responsive genes, such as *MYB107*, *CAX3*, *POX1*, *MT3*, and *PCS1*, suggesting that H_2_S and CDPK are linked under Cd stress [114].

All of these results show that H_2_S and Ca^2+^ signaling, especially under adverse conditions, are interrelated. Ca^2+^ signaling can activate LCD enzyme activity, thereby promoting the accumulation of H_2_S. In turn, H_2_S regulates the expression of stress response-related genes by stimulating the Ca^2+^ signal. Together, these two signals enhance the tolerance of the plant to stress.

#### 3.3.2. Crosstalk of H_2_S with Na^+^ and K^+^

Salt stress invariably causes a rapid increase in the intracellular Na^+^ level and leads to an imbalance of Na^+^/K^+^, which in turn represses plant growth. Therefore, maintaining the balance of Na^+^/K^+^ is a crucial factor in conquering salt stress [115]. Several studies have proclaimed that H_2_S can reduce the sensitivity of plants to salt stress mainly by preventing both uptake of Na^+^ and K^+^ efflux and by promoting Na^+^ efflux and uptake of K^+^ and thus mediating the balance of Na^+^/K^+^ [116,117,118], which have begun to reveal the regulatory mechanisms by which H_2_S helps to mediate the balance of Na^+^/K^+^. In wheat, the addition of CaCl_2_ (an inhibitor of nonselective cation channels (NSCCs)) or amiloride (an inhibitor of salt overly sensitive 1 (SOS1), a Na^+^/H^+^ antiporter) disrupts the Na^+^/K^+^ balance promoted by H_2_S, indicating that NSCC and SOS1 may be the main pathway of reducing Na^+^ by H_2_S [116]. In *Populus popularis*, NaCl induces K^+^ loss mainly due to the activation of H^+^-ATPase on the plasma membrane. Application of Na^+^/H^+^ antiporter inhibitors, sodium orthovanadate and amiloride effectively inhibited the Na^+^ efflux, but NaHS enhanced it. Thus, the Na^+^/K^+^ balance maintained by H_2_S may be achieved by regulating the Na^+^/H^+^ antiport system in *Populus popularis* [117]. In Arabidopsis, application of NaHS alleviates the suppression of salt stress on root growth and promotes the accumulation of H_2_O_2_, while exogenous application of H_2_O_2_ reduces the ratio of Na^+^/H^+^ and strengthens the role of H_2_S. Application of a ROS scavenger (DMTU), a plasma membrane (PM) NADPH oxidase inhibitor (DPI) or a glucose-6-phosphate dehydrogenase (G6PDH) inhibitor (glycerol) all eliminate the effect of H_2_S, further indicating that H_2_O_2_ may be involved in the H_2_S-mediated tolerance to salt stress via the regulation of G6PDH and PM NADPH oxidase [119]. In conclusion, under salt stress, H_2_S works to maintain ion homeostasis within plant cells by regulating the Na^+^/H^+^ antiport system in the way that is H_2_O_2_-dependent and that uses the enzymes NSCCs and the SOS1 antiporter to reduce Na^+^ levels.

### 3.4. S-sulfhydration Modification of Proteins Mediated by H_2_S

At present, many studies have proved that H_2_S can regulate the spatial structure of certain target proteins via the post-translational modification named S-sulfhydration. S-sulfhydration affects protein structure, subcellular localization, and function, in a way that can regulate plant growth and development and responses to stress [65]. S-sulfhydration occurs when H_2_S reacts with Cys residues (-SH, -S-S-, -S-OH or S-NO) in target proteins to form a persulfide group (-SSH) [120]. In a persulfidation proteome in Arabidopsis treated with NaHS, a total of 106 persulfidated proteins were identified, which were mainly involved in photosynthesis, protein synthesis, cell organization, and primary metabolism [100,121]. Using a different technique, proteome analysis of endogenous persulfidated proteins in leaves of WT Arabidopsis and the *des1* mutant identified 2015 persulfidated proteins, which were mainly involved in regulating primary metabolism, responses to abiotic and biotic stress, plant growth and development, and RNA translation [65]. At least 5% of proteins in Arabidopsis may be persulfidated under normal growth conditions [65], which is consistent with the persulfidation proteome with application of NaHS [100]. Further analysis found that the activities of APX, glyceraldehyde 3-phosphate dehydrogenase (GAPDH) and glyceraldehyde 3-phosphate dehydrogenase, isoform C1 (GAPC1) were increased by S-sulfhydration, indicating that S-sulfhydration may be a mechanism that promotes reduction of oxidative stress in plants [100]. Physiological research further confirms that S-sulfhydration, mediated by H_2_S, plays key roles in plant growth, development, and stress response. For example, Li et al. found that ACTIN2 (ACT2) can be S-sulfhydrated by H_2_S at Cys287. This S-sulfhydration interrupts actin-2 polymerization, resulting in root hair dysplasia in Arabidopsis [19]. Furthermore, Shen et al. (2020) and Chen et al. (2020) found that ABA-induced stomatal closure was also related to H_2_S-mediated S-sulfhydration. In Arabidopsis, ABA addition stimulates the S-sulfhydration of DES1 at Cys44 and Cys205 to activate DES1, which catalyzes the accumulation of H_2_S [55,56]. This higher levels of H_2_S then mediates the S-sulfhydration of SnRK2.6 at Cys131 and Cys137, promote its activity and the interaction between SnRK2.6 and ABF2, which in turn positively regulates ABA signaling [56]. On the other hand, the produced H_2_S also drives the S-sulfhydration of RBOHD at Cys825 and Cys890, enhancing the production of ROS. Physiologically, ROS is the rate-limiting messenger in ABA-mediated stomatal closure and is part of the negative feedback loop for inhibiting ABA signal [55].

In cucumber, H_2_S improves cold tolerance via actively modifying the synthesis of Cucurbitacin C (CuC) by driving S-sulfhydration of the His-Csa5G156220 and His-Csa5G157230 proteins, transcription factors that activate the CuC synthetase gene [122]. In tomato, H_2_S, as a downstream component of ET-induced stomatal closure, reduces ET content by impairing the activity of ACOs through persulfidation, which in turn enhances the osmotic stress response [50]. Consequently, H_2_S-mediated S-sulfhydration occurs during many aspects of plant growth and S-sulfhydration of proteins may be an essential mechanism by which H_2_S affects plant growth and development under both normal and stress conditions.

Based on the above descriptions, it can be clearly seen that H_2_S does not function independently in plants, but interacts with plant hormones and other signaling molecules, such as Ca^2+^, NO, H_2_O_2_, and even proteins, form a complex signaling network that finely regulates plant growth, development, and stress responses. In the future, we can make full use of advanced proteomics to further explore the mechanisms by which H_2_S influences signaling pathways in plants.

## 4. Conclusions and Perspectives

**Figure 2:**
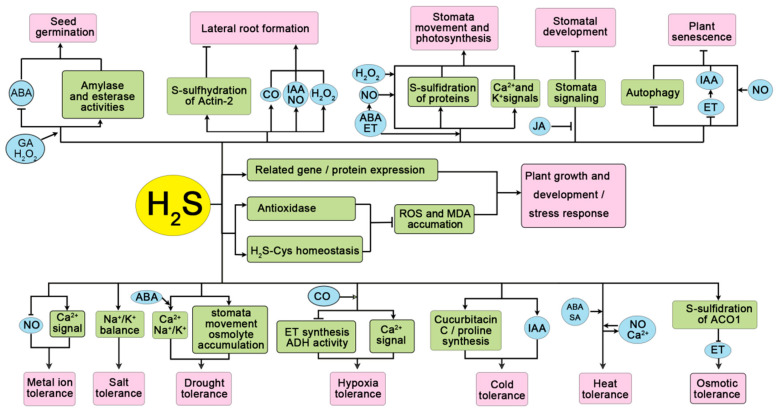
A model of the roles of H_2_S in plant development and stress responses. H_2_S has recently been recognized as a novel gaseous signaling molecule with various functions during plant development at different stages and during stress responses. H_2_S functions by promoting the expression of specific genes, enhancing the activity of the antioxidant system and maintaining H_2_S-Cys homeostasis. Growing evidence suggests that H_2_S is involved in seed germination, by increasing amylase and esterase content for greater energy efficiency. H_2_S can also fine-tune lateral root formation, stomatal movement, photosynthesis, and plant senescence by regulating protein S-sulfhydration and by establishing crosstalk with CO, NO, IAA, ABA, ET, and other signaling pathways. In addition, H_2_S may also be involved in plant senescence by inhibiting autophagy. Both exogenous and endogenous H_2_S are able to optimize plant adaptation to various stresses (e.g., metal ion, drought, hypoxia, temperature, salt, and osmotic stress) through positively regulating ionic equilibrium, stomatal movement, osmolyte accumulation, ethylene synthesis, related enzyme activity, interaction with other reactive species, and plant hormones. H_2_S can also regulate the expression of related genes and proteins, reduce the oxidative stress caused by various stresses by enhancing the activities of antioxidant enzymes and the accumulation of antioxidants, so as to improve the stress resistance and promote plant development.

Continuing investigation into H_2_S has revealed its numerous and varied regulatory roles in biology and has brought more attention to this gasotransmitter. It is now recognized that H_2_S promotes seed germination, root development, photosynthesis, stomatal movement, and plant senescence. H_2_S also regulates plant responses to stress by activating antioxidant defenses, improving expression of genes encoding resistance-related enzymes, and interacting with different signaling molecules. Additionally, S-sulfhydration of proteins induced by H_2_S is an essential mediator (Figure 2).

However, there remain numerous issues to be explored. For example, it has been confirmed that an appropriate concentration of H_2_S produces a marked effect on plant development and responses to stress, but different plants have different tolerances to H_2_S. This means it is particularly important to monitor the concentration of H_2_S in cells. Second, most of the existing research has focused on how exogenous H_2_S improves plant resistance to stress, but the mechanism(s) by which endogenous H_2_S functions is barely clear. In some studies, the *des1* mutant exhibited stronger tolerance to Cd and pathogen stress, which differs from the theory that increases in H_2_S could improve the stress resistance of plants. Therefore, it is not clear whether H_2_S enhances the antioxidant capacity of plants through the homeostasis of H_2_S-Cys or as an antioxidant signaling molecule itself. Furthermore, it is unclear if endogenous and exogenous H_2_S have different function mechanisms. It is also unknown how environmental stimulation triggers the accumulation of H_2_S, how cells perceive the H_2_S signal and what are the direct targets and downstream cascades of H_2_S plant signal transduction. Numerous reports have documented that H_2_S can crosstalk with the signaling pathways of plant hormones, other gasotransmitters, and ions to form a complex regulatory network for all aspects of plant growth and development, but the interactional mechanisms of H_2_S with other signals remain to be elucidated. It is also unknown whether H_2_S plays important roles through its receptor. Therefore, the functions of H_2_S in plant growth and development need to be deeply studied by transcriptomics, proteomics, metabolomics, and functional genomics, in combination with more genetic materials and H_2_S donors, scavengers, and synthetic inhibitors in the future.

## Abbreviations

S	Sulfur
Cys	Cysteine
L-Cys	L-cysteine
Met	Methionine
H_2_S	Hydrogen sulfide
SIR	Sulfite reductase
OAS-TL	O-acetylserine (thiol) lyase
OAS	O-acetylserine
APS	Adenosine 5′-phosphosulfate
ATPS	ATP sulfurylase
APSR	Adenosine- 5′- phosphoryl sulfate reductase
CDes	Cysteine desulfhydrase
LCD	L-cysteine desulfhydrase
DES1	L-cysteine desulfhydrase 1
DCD1	D-cysteine desulfhydrase1
DCD2	D-cysteine desulfhydrase2
CAS	β-cyanoalanine synthase
CN^−^	Cyanide
GSH	Glutathione
AOA	Aminooxyacetic acid, an H_2_S synthesis inhibitor
HT	Hypotaurine, an H_2_S scavenger
PAG	Propargylglycine, a DES1 inhibitor
NPA	N-1-naphthylphthalamic acid, IAA transport inhibitor
Cptio	2-(4-carboxyphenyl)-4,4,5,5-tetramethylimidazoline-1-oxyl-3-oxide, NO scavenger
GSNOR1	S-nitrosoglutathione reductase 1
GAPDH	Glyceraldehyde phosphate dehydrogenase
SNP	Sodium nitroprusside, NO donor
HA	Hydroxylamine, H_2_S synthesis inhibitor
